# Healthy Water-Based Tourism Experiences: Their Contribution to Quality of Life, Satisfaction and Loyalty

**DOI:** 10.3390/ijerph17061961

**Published:** 2020-03-17

**Authors:** Ana María Campón-Cerro, Elide Di-Clemente, José Manuel Hernández-Mogollón, José Antonio Folgado-Fernández

**Affiliations:** 1 Department of Business Management and Sociology, School of Business Studies and Tourism, University of Extremadura, Avda. de la Universidad s/n, 10071 Cáceres, Spain; jmherdez@unex.es; 2 Department of Business Management and Sociology, Research Institutes, LAB 0L3, University of Extremadura, Avda. de las Ciencias s/n, 10004 Cáceres, Spain; ediclemente@unex.es; 3 Department of Financial Economics and Accounting, School of Business Studies and Tourism, University of Extremadura Avda. de la Universidad s/n, 10071 Cáceres, Spain; jafolgado@unex.es

**Keywords:** natural settings, water-based experiences, health tourism, quality of life, destination marketing

## Abstract

The scientific literature on tourism identifies two driving trends: the quest for experientiality and the growing connection between holidays and quality of life. The present research focuses on water-based activities practiced with a healthy purpose, capable of driving positive economic, social and environmental effects on the territory where this type of tourism is developed. Considering the growing demand of experiential tourism, it is important to assess the experiential value of these practices and their impact on the quality of life, satisfaction and loyalty. A sample of 184 customers of thermal spas and similar establishments was used to test the structural model proposed, employing the partial least squares technique. The results show the experiential value of healthy water-based activities and confirm their positive impact on the individuals’ quality of life, satisfaction and loyalty towards both the experience and the destination.

## 1. Introduction

The tourism industry is undergoing a substantial change. The advance in new technologies and a skilled and demanding consumer target means that the organisations and destinations need new marketing and management tools to meet the modern tourists’ expectations and the industry’s requirements for innovation [1].

The scientific literature on tourism issues identifies two driving trends: the quest for experientiality and the growing connection existing between holidaymaking and perceived improvements in individuals’ quality of life. The former is forcing the tourism sector to face a new competitive scenario. Increasing importance is being given to the emotional value of the tourism experiences offered, leaving in the background their functional properties [2]. The latter is a facet of the tourism phenomenon which is gaining momentum in recent times. Tourism literature has shown a growing consensus about the benefits that individuals can get from tourism experiences and meaningful travel [3,4,5,6].

In this new experiential stream, tourism businesses face the challenge of changing their business and commercial strategies and improving the affective components of their products, that is, being able to deliver pleasant sensations and memories to the consumer, as well as ensuring the practical functionality of the goods/services offered [7]. The functional qualities of a tourism proposal are no longer considered differentiators and are not enough to capture the attention and the preferences of consumers.

Considering the preceding, the tourism industry is in need of drawing innovative tourism proposals in line with the recent requirements of modern tourists who see in holidaymaking a possible avenue to pursue happiness [8]. However, still very little is known about how tourism contributes to quality of life and whether some specific practices are more suitable than others to turn holidays into significant enhancers of personal happiness [9]. 

According to Nawijn [10], ‘in the light of the experience economy’ (p. 560), the tourism industry could improve its performance and foster the effect that holidays have on happiness, giving more attention to the experiential content of the tourism offered, and thus, understanding what causes happiness. The author realises that certain types of holidays are worth further examination to this extent, as they show the potential to positively impact tourists’ happiness. The ones he suggests are wellness tourism, promulgating physical and psychological recovery, or slow tourism, suggesting people should travel with slower means of transport in order to enjoy the trip and experience relaxed rhythms. Based on this consideration, the present research focuses on water-related activities practiced with a healthy purpose, that besides their experiential potential due to the sensorial properties of water (unique touch, sounds, flavours, colours, cold or hot feelings, etc.), accomplish the objective of preserving water resources and ecosystems from contamination, which is often the case of the touristic use of water [11,12]. In fact, natural water resources, such as rivers, lakes, streams, wetlands, aquifers and estuaries, are often jeopardised by people’s use, forcing a change in water policies and management [13].

Water is for sure a resource with an inestimable environmental value, which unfortunately is not sufficient to preserve it from misuses. Draper [14] points out that a wise management of water resources needs a commitment with a dynamic economy, social equity and healthy environment. Even if tourism is traditionally considered a water-consuming industry whose impact on its conservation is usually assessed to be more negative than positive, it is possible to encourage a proper and sustainable tourism use of water resources. 

Tourism can contribute to this objective by means of giving to intangible water heritages a tangible value, that is, an economic (besides environmental) value in order to save it and its connected water-based ecosystems and biodiversity from destruction. Water-based experiences are potential solutions to preserve both the environmental and the economic value of water, and its tangible and intangible heritage [15]. However, Essex et al. [16] claim that ‘there is little research on the significance of water in tourism development’ (p. 6). In the same line, Jennings [17] maintains that the theme of water-based experiences in tourism has been little explored. Therefore, this work turns the spotlight onto tourism experiences based on water natural resources and settings with the aim of exploring their touristic value within the new experiential context. 

Luo et al. [18] assert that experience economy research has not focused on the customer experience in wellness tourism, and also claim that it is relevant to understand how visitors achieve a bettering of their quality of life through this type of tourism.

The most renowned water-based activities related to health are the visits to thermal spas and similar establishments (spas, Arab baths, etc.) [17]. However, there are other activities that involve water as a key element, and these are the enjoyment of landscapes and soundscapes related to water, visiting fluvial beaches, trekking through routes with water resources, enjoying river boat trips and watching aquatic birds. Moreover, drinking mineral and medicinal waters provides significant benefits for human health. Restaurants have begun offering a water menu along with the wine menu. The recent concern about health and wellbeing that characterises modern society involves water as a functional element, where a conscious consumption can enhance personal wellness. This trend contributes to generating a new ‘water culture’ [19] that can lead to the development of innovative initiatives in the tourism sector, addressed to those consumers who will benefit from water properties in places where this element is a central attraction. The rise of a new ‘water culture’ can have beneficial effects on both individuals’ health and on tourism destinations’ economies. 

Therefore, it is important to assess the potential of these tourism experiences linked to water and health and their impact on outcome variables such as the tourists’ satisfaction, loyalty and quality of life. Water-based experiences can generate long-term revenue, driving positive behavioural intentions. The main objective of this research is to analyse water-based tourism experiences as a strategy capable of fostering the tourists’ satisfaction, quality of life, and positive future behaviours. As a consequence, water-based experiences could be assumed as capable of enhancing the economic, social and environmental sustainability of a destination where singular hydrographical resources are placed. This work tries to assess tourism activities based on binomial water-health through the experience model proposed by Pine and Gilmore [20]. This is an original contribution to tourism research as it is the first attempt: (1) to obtain an integrative perspective about the phenomenon of tourism experiences based on water and health; (2) to offer new ideas for tourism products development using water in a non-consumptive way, in line with the modern tourists’ demand of experientiality and wellbeing; (3) to test whether water-based activities accomplish the objective of enhancing individuals’ quality of life which, in turn, can contribute to driving tourists’ loyal behaviours. 

The results achieved offer insightful ideas for the elaboration of new experiential proposals and show marketers and practitioners the most suitable actions to undertake in order to satisfy current tourism demand, expecting travel to be a changing and once-in-a-lifetime experience, using water as a tourism attraction from an environmentally respectful perspective.

## 2. Materials and Methods

### 2.1. Experiential Tourism: The Experience and Its Dimensions

The theoretical background that supports the hypotheses’ definition and operationalization of the concepts involved in this research has to be seen in the theory of experience economy and its application in the tourism industry. The rise of the experiential trend in modern economies is nowadays bringing the hospitality and tourism sector into a new competitive stage. New technological advances and the easy access to information have meant that those elements traditionally designed to differentiate what is being offered in the market can be easily replicated by competitors, nullifying their differential power and making them interchangeable in consumers’ eyes [21]. According to Jensen and Prebensen [22], experience-based tourism can be considered an offer that differentiates itself from more conventional tourism practices due to its intangible and emotional value which is what modern tourists seek and appreciate most in their holiday time. Therefore, the tourism industry faces the challenge of turning its proposals into experiences and providing what is currently being offered with a new emotional and intangible value in order to identify new competitive advantages [23].

Water and tourism have traditionally been studied from two different focal points: the environmental concern, as tourism activities are water-consuming and polluting practices [11]; and the health and wellness perspective, with a specific reference to thermal and spa activities [24,25]. This work considers the water-tourism connections under this second approach and highlights the experiential value of water-related tourism practices. 

Health and wellness tourism has recently been a focus of attraction in tourism research as, nowadays, people are particularly sensitive to safeguarding their personal wellness and conducting a healthy lifestyle [3]. As a consequence, the concept of health has widened its boundaries, being a synonym for happiness, wellbeing and long life, and more than just the absence of diseases [25]. This new social consciousness introduces some changes regarding tourists’ decisions and preferences and offers some new opportunities for tourism destinations’ management and innovation. 

Besides rest and relaxation, physical and psychological recovery, modern tourists have an expectation of personal enrichment in their holidays [26]. In this line, experiential travel and healthy practices may provide benefits to tourists beyond satisfaction and enjoyment [4], contributing to enhancing their perception of personal quality of life [8,9].

Pine and Gilmore [20] made the most significant contribution to the definition of the experience concept. The authors developed a conceptual model defining experiences which has been largely applied to assessing experientiality in different tourism contexts [27,28,29]. The authors describe experiences by means of two dimensions: participation and connection, embedded in a spectrum from passive to active, and from absorption to immersion. The participation implies that consumers are impacted by the experience, while the connection dimension assesses the degree to which the tourists themselves impact the experience, contributing to its creation [20]. The intersection of these two dimensions gives birth to four realms defining the experience concept, also known as the 4Es’ model, as its components are entertainment, education, esthetics and escape. 

The water-related activities considered in this research have a high experiential potential. At a conceptual level they fit the model proposed by Pine and Gilmore [20]. With regard to the ‘escape’ component, these activities are often held in outstanding natural settings which induce the feeling of escaping from the daily contexts. The physical contact with water has a relaxing power due to the feeling of the lack of body weight perceived while immersed. Therefore, tourists who decide to practice these activities have, in water, an effective vehicle to escape from stress and routine. The ‘esthetics’ dimension is provided by the beauty of the landscapes related to water. Healthy water-related activities are ‘educational’ as tourists who choose these practices can learn about the beneficial properties of water for human health and differentiate between the kinds of waters and the effects of its uses. Finally, water-based experiences provide ‘entertainment’ with activities such as observing landscapes, the contemplation of sounds and the colours of water, and the enjoyment of baths and water-treatments.

### 2.2. Variables and Hypotheses Definition

#### 2.2.1. Experiential Satisfaction

According to Kim et al. [30], research on travel and tourism has largely examined the tourists’ satisfaction concept. Similarly, Neal and Gursoy [31] assert that customer satisfaction is frequently examined for being a topic capable of enhancing the destination’s competitiveness by means of inducing loyal behaviors and intentions of revisiting the destination in the future [32].

The new experiential push that pervaded the tourism industry, as well as the whole modern economy, entailed some changes in the treatment of satisfaction. This variable has been traditionally considered to be predicted by functional factors (i.e., quality, value and image) [30,33]. However, few researches offer useful insights demonstrating that new affective and emotional concepts, such as pleasure, arousal, joy, love, positive surprise, mood and hedonics, are gradually integrating [7,34,35] or even substituting the traditional utility-based approach to satisfaction [7,36,37]. Satisfaction is considered a key driver for customer experience assessment [38].

Scientific literature provides numerous evidences supporting the relationship between emotions and satisfaction [36] and shows that a growing consensus exists on the need to incorporate emotional and affective components in the assessment of this variable [39]. Lin and Kuo [40] found proof of the relationship between tourist experience and satisfaction. Agyeiwaah et al. [41] demonstrate the relationship in the context of culinary tourism, Ali et al. [42] in creative tourism, and more specifically, Luo et al. [18] in wellness tourism experiences. 

According to Pine and Gilmore [43] the 4Es’ model leads to satisfaction. Some researchers have already tested the relationship between the experience concept and satisfaction. Oh et al. [29] found significant evidence linking the esthetic component of the experience and satisfaction. Similarly, Hosany and Witham [28] demonstrated that esthetics and entertainment significantly contribute to satisfaction. Quadri-Felitti and Fiore [27] empirically showed the positive relationship between education and esthetics on satisfaction. Considering the preceding, the following hypothesis is proposed:

**Hypothesis** **1.** **(H1):** Healthy water-based experiences have a positive impact on tourists’ experiential satisfaction.

#### 2.2.2. Quality of Life

Holidays are generally considered events that increase wellbeing and quality of life [44]. Research on quality of life regarding the tourism experience is an emerging area of study, considered as an important field of tourism studies because of its relationship with short-term and long-term effects on individuals, on businesses, and on society [38]. According to Luo et al. [18] since the past decade, wellness tourism has been a booming industry, making it relevant to understand how visitors achieve quality of life through the wellness experience, in which healthy water-based tourism experiences has to be included. 

Connections between tourism and quality of life have started to be explored and have recently become a focus in tourism studies [45]. Many authors started to test the potential relationship that exists between tourism experiences, travellers’ satisfaction and tourists’ happiness [3,8,9,46,47]. Gilbert and Abdullah [48] suggest that holidaymaking can improve the level of happiness experienced by tourists. Similarly, Puczkó and Smith [49] define holidays as ‘a state of temporary happiness’ (p. 265) associated with some specific activities and behaviours that people have while on holidays. 

Neal et al. [46] in their study on vacation experience and quality of life showed that satisfaction with tourism services is a positive determinant of overall quality of life. More specifically, within the experiential context, Kim et al. [26] confirms that a strong relationship links satisfaction with a travel experience together with the individuals’ perception of their overall quality of life. Luo et al. [18] found that in wellness tourism visitors’ satisfaction with experiences predict quality of life. This supports the following hypothesis:

**Hypothesis** **2.** **(H2):** Experience satisfaction has a positive impact on tourists’ quality of life.

#### 2.2.3. Loyalty

Loyalty is a traditional marketing outcome where the importance has been increasingly recognised in tourism and hospitality research [50]. Satisfaction is often considered a significant determinant of loyalty and future behaviour intentions [51,52]. It could be thought that providing satisfying experiences will possibly drive loyal behaviours in the future, which usually coincides with positive word-of-mouth and revisiting intentions. However, the tourism market is in constant change and new trends in consumers’ desires and needs bring tourism marketing to face ever-new challenges that make it more difficult for the plain satisfaction-loyalty binomial to remain effective. Kim and Ritchie [53] maintain that satisfaction alone is no longer enough to drive positive future behaviours, as researches have noted that more than the 60% of satisfied costumers decide to switch to another firm. Thus, it has to be recognized that, in order for satisfaction to effectively result in loyal intentions, some other components should intervene. 

Experientiality is challenging the traditional idea of loyalty [54,55]. The tourists’ search for unique experiences and wanderlust are forcing a reassessment of the concept in light of new experientiality. In this context, loyalty should be, on one hand, addressed towards new experience-related objects, rather than the destination (i.e., the kind of experience itself), and on the other, new antecedents should be involved in the loyalty-forming process (i.e., quality of life). As a consequence, this research considers loyalty towards two objects: the destination and the water-based experiences. In addition, quality of life is introduced in the conceptual model as an antecedent of both variables considered for loyalty. 

Within the experiential literature, some studies confirm that experiential satisfaction is a direct antecedent of behavioural intentions [56,57,58] and that quality of life, or similar concepts, is a new antecedent of loyalty [26,54,59].

Literature points out that positive tourism experiences could enhance repeat visits and recommendations [41]. Wu and colleagues [56,57,58] provide empirical evidences supporting the theoretical relationship that links the experiential satisfaction with loyalty. The authors in their studies on theme parks [58], the golf industry [56] and heritage tourism [57] confirm that experiential satisfaction leads to loyal behaviours in the future. Other authors in other tourism contexts verified that relationship [40,41,42]. These results offer a valuable support to the following hypotheses:

**Hypothesis** **3.** **(H3):** Experiential satisfaction has a positive impact on loyalty to the experience.

**Hypothesis** **4.** **(H4):** Experiential satisfaction has a positive impact on loyalty to the destination.

With regard to the consideration of quality of life as a direct antecedent of loyalty, some valuable insights can be found in Kim et al. [26], Lin [54] and Kim et al. [59].

Kim et al. [59], in their study on chain restaurants, confirm that consumers’ wellbeing perceptions are the most powerful antecedents of future positive behaviours. Lin [54] shows that cuisine experiences and psychological wellbeing are important determinants of revisit intentions. Kim et al. [26], following a structural path starting from elderly tourists’ involvement in tourism experiences and resulting in revisiting intentions, showed how satisfaction and quality of life contribute to determine the tourists desire to revisit the destination. Their results confirm that quality of life is an effective predictor of loyal behaviours. Therefore, the following hypotheses are proposed:

**Hypothesis** **5.** **(H5):** Quality of life has a positive impact on loyalty to the experience.

**Hypothesis** **6.** **(H6):** Quality of life has a positive impact on loyalty to the destination.

**Figure 1:**
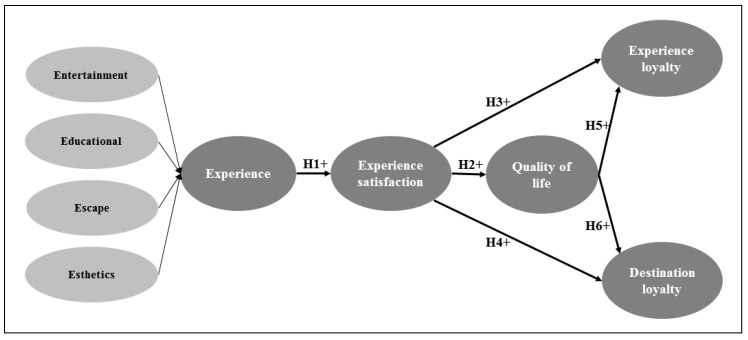
Theoretical model.

The hypothesized relationships are graphically presented in Figure 1. 

## 3. Methodology

The study setting is the region of Extremadura, a southwest region of Spain, where water has been traditionally seen as an abundant resource. However, growing irrigation demands under a low price are outstripping the supply of raw water and competing with other uses [60], a challenge that could be faced with sustainable tourism practices. The region relies on more than 1500 kilometres of inland water, more than 60 natural-based bath areas and 7 thermal spas, according to the Touristic Plan of Extremadura 2017–2020 promoted by the Government of Extremadura. The data shapes the region as a destination to enjoy diverse tourism water experiences, and thus an excellent place to locate this study. 

This research relied on an exploratory study using quantitative methodology to evaluate the proposed model through analyses based on structural equation modelling (SEM), due to its capacity to test several relationships established in a model that emerges from theory [61]. 

The population was identified in customers of thermal spas of Extremadura and other similar establishments. The tool used for data collection was a self-administered paper-based survey, complemented with an online survey.

The scales used to measure the variables of the model were validated in previous studies and have been adapted to the context of this research (see Table 1). 

The questionnaire used multi-item scales rather than one-item scales, as suggested by MacKenzie et al. [63]. The indicators were measured on a five-point Likert scale.

The questionnaire was distributed to the customers enjoying thermal spa visits and other similar water-based experiences in Extremadura. The dissemination of the questionnaires was conducted using two procedures: a paper-based questionnaire in thermal establishments and an online questionnaire. To ensure that no biases were introduced in data analysis due to the use of two collecting procedures, a t-test for independent samples was performed. The results confirm the equality of means between the two groups of data. Only 2 out of 32 indicators showed statistically significant differences, thus the potential bias was minimal and can be assumed. The two subsamples have been unified for the model assessment. 

A total of 184 completed questionnaires were collected between 3th of November and 24th of December of 2017, using a non-probability convenience sampling. The sample size is suitable as it accomplishes the criterion proposed by Hair et al. [64], who propose a minimum value for the item-response ratio between 1:5 and 1:10.

Following Hair et al.’s [61] guidelines, the partial least square (PLS) technique was considered the most appropriate method for the assessment of the hypothesised model versus models based on covariances, considering that it contains a second-order construct (experience) and reflective and formative indicators, thus a complex model structure. It is also appropriate for relatively small samples, as in this study. In addition, the PLS algorithm transforms non-normal data, so results are robust to the condition of normality [65]. The SmartPLS 2.0 M3 software (SmartPLS GmbH, Hamburg, Germany) was employed for the model evaluation, while the descriptive analysis and the collinearity test were performed with IBM SPSS Statistics, Version 22 (IBM Corp, Armonk, NY, USA)

## 4. Results

### 4.1. Sample Profile

The sample was composed of 37.0% men and 57.6% women. Within this research, the tourists visiting thermal spas in Extremadura (Spain) came from other Spanish regions (56.7%), had a mature age (59.8% were ‘more than 55 years old’) and a high education level (36.4%) (see Table 2). 

Regarding the frequency that this sample engages in tourism experiences linked to the binomial water-health, 34.2% asserts that they only ‘sometimes’ practice this kind of activity, and 31.0% said ‘frequently’. This result points out that the users of thermal spas surveyed are loyal to tourism water-related experiences. With respect to the interest that respondents have in water-based experiences, the most valued one was ‘visiting health spas’ (3.87 out of 5 points), followed by ‘observing landscapes related to water’ (3.68), ‘visiting fluvial beaches’ (3.41) and ‘trekking routes related to water’ (3.35). It is important to highlight that the other rated activities, which were ‘visiting spas’, ‘river boat trips’, ‘watching aquatic birds’ and ‘asking for water menus in a restaurant’ have achieved a mean over 2.5. Respondents recognise the benefit that water-based tourism experiences have on health, considering the high scores registered by this indicator (4.24 out of 5). The sample also confirms a high interest in including water-based experiences in their trips (4.01). Hence, these activities reckon with the potential of a latent tourism demand. In addition, respondents appreciate water-based tourism experiences as potential enhancers of personal health (4.01) (see Table 3).

### 4.2. Analysis of The Model

#### 4.2.1. Measurement Model Assessment

Since the proposed model is multidimensional, the two-stage approach was selected for its assessment [66]. In order to perform the model assessment, this study followed the guidelines proposed by Wright et al. [67]. Following MacKenzie et al.’s indications [63], the constructs taken into account in the first step were considered reflective. Consequently, the measurement model was evaluated to assess the items’ reliability, internal consistency, convergent validity and discriminant validity [61]. Regarding individual reliability, all the indicators are above the acceptable threshold of 0.707 [61,68]. Construct reliability was measured through composite reliability (CR). According to Nunnally and Bernstein [69], the values obtained in this study are acceptable, being in the range of 0.60–0.70 (see Table 4). 

With regard to convergent validity, the average variance extracted (AVE) is above 0.50, so all the constructs fall into the adequate parameters according to Hair et al. [61] (see Table 4). Discriminant validity is confirmed when the correlations between the constructs are lower than the square root of the AVE (values in bold in Table 5) [68]. 

The scores resulting from the first step can now be used for the second step in order to model the second-order construct (experience). The aggregated scores, calculated by PLS for the experience construct, generate a new set of data to be used in the following analysis. The model shows a novel nomological structure, including reflective and formative variables, that needs to be assessed in its measurement and structural validity. The experience construct now acts as formative [63]. Moreover, the dimensionality of experience, proposed by Pine and Gilmore [20], has been widely confirmed in previous research and this is assumed to be further evidence supporting the formative nature of the construct.

The reflective measurement model was analysed by repeating the steps described above. The analysis of items’ reliability, CR and AVE revealed a satisfactory evaluation (see Table 6). 

Table 7 shows that discriminant validity is demonstrated.

The evaluation of a formative measurement model required an examination of any possible multicollinearity between the indicators, an assessment of the weight of each indicator and a review of their significance. For all the indicators, the variance inflation factor (VIF) was below 5 [61]. Therefore, no problem was found with multicollinearity between the indicators of the experience construct. The weights of the indicators entertainment and escape are statistically significant. However, the weights of the indicators educational and escape are not significant. Nevertheless, some authors recommend maintaining the items, as long as their loadings are statistically significant, at a confidence level of 99%, and absence of multicollinearity is assured [70] (see Table 8). 

#### 4.2.2. Structural Model Assessment

R^2^ of each dependent construct needs to be analysed, as well as the paths’ significance, by using the bootstrapping method [61]. Table 7 shows the R^2^ values for the endogenous variables. The best explained variable is experience loyalty (67.1% or substantial-moderate), followed by destination loyalty (64.7% or substantial-moderate), experience satisfaction (51.9% or moderate) and, finally, quality of life (46.0% or moderate). This table also shows how much the predictive variables contribute to the explained variance of the endogenous variables [71]. The analysis of the structural paths’ significance was done with the bootstrapping method, following Hair et al.’s [61] guidelines. All the hypotheses are statistically significant (see Table 9).

**Figure 2:**
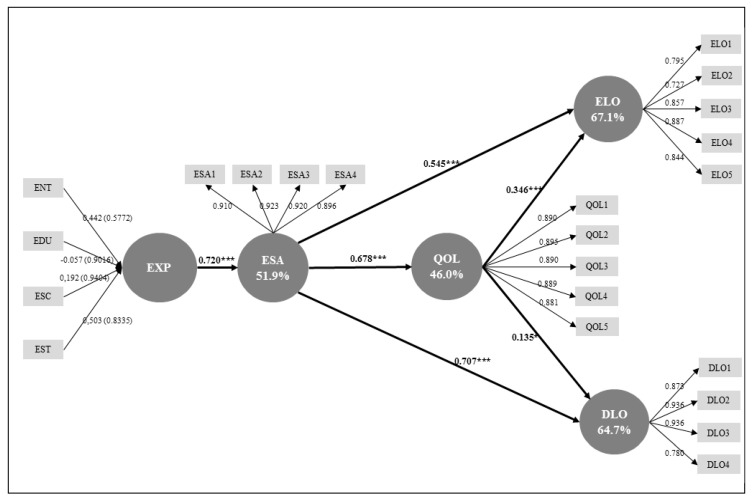
Graphical summary of the model assessment. * ENT: Entertainment. EDU: Educational. ESC: Escape. EST: Esthetics. EXP: Experience. ESA: Experience Satisfaction. QOL: Quality of life. ELO: Experience loyalty. DLO: Destination loyalty.

Figure 2 graphically presents the results of the measurement and structural model assessment.

## 5. Discussion

The model proposed in this study provides a better comprehension of the impact of healthy water-based tourism experiences in perceived quality of life, satisfaction and loyalty. It has been empirically validated supporting all the model hypotheses established from the literature review. Thus, the results show the importance of offering experiential value with tourism products in the context of tourism experiences based on water and health. The model offers a substantial-moderate capacity to explain the variation of the endogenous variables, which are experience satisfaction (R^2^ = 51.9%), quality of life (R^2^ = 46.0%), experience loyalty (R^2^ = 67.1%) and destination loyalty (R^2^ = 64.7%). It is worth noting the role of experience satisfaction in determining experience loyalty (42.4%) and destination loyalty (56.4%). The positive relationship between satisfaction and loyalty has been largely confirmed in scientific literature [51,52] and it is further proven in the context of healthy water-based tourism experiences. 

It is important to highlight the positive impact that the experience variable exerts on experience satisfaction (β = 0.720 ***) (H1+). This result is consistent with past research [40,41,42,43], and more specifically with Luo et al.’s [18] research that verified the relationship between wellness tourism experience and satisfaction. 

An assessment of the dimensionality of the experience reveals that entertainment and esthetics are the most determining factors of the construct. This result is closely related to the outcomes reached by Oh et al. [29] and Hosany and Witham [28] who found a key role of these components versus education and escape. Similarly, Quadri-Felitti and Fiore [27] identified the impact of esthetic on satisfaction. According to Oh et al. [29], these findings suggest the relative importance of the four realms of the experience proposed by Pine and Gilmore [20] depending on the study context. The results of this research, showing the remarkable relevance of the entertainment and esthetics dimensions, is reasonable if considering that baths and water treatments are enjoyable practices and that water with its shapes, sounds, movements and colours embellishes outdoor and indoor spaces. 

The education and escape dimensions turned out to be of lower importance. Regarding the former, an explanation exists that can be found in the low consciousness about the properties that water has on human health, and a general lack of ‘water culture’. The latter can be explained by the therapeutic focus of many thermal spas and similar establishments. This can introduce the feeling of being involved in medical practices to address health problems or ailments more than in pleasant activities capable of taking tourists away from daily problems and worries. This may suggest the importance of complementing the traditional thermal spas’ offerings with new proposals related with the enjoyment of water and more focus on wellbeing rather than on health. In short, education and escape may become more important dimensions when a water culture has gained force. 

Even if statistics do not fully support the role of education and escape as determining factors of the experience construct as their weights turned out to be non-significant, they still make a contribution to the definition of the experience variable according to their loadings’ scores (see Table 6). Therefore, they cannot be disregarded.

Regarding the link between experience satisfaction and the individual’s quality of life (H2+), the findings of this study are consistent with the ones obtained by Neal et al. [46] and Kim et al. [26]. The results also confirm the strength of this relationship (β = 0.678 ***), concluding that healthy tourism water-based experiences are effective enhancers of quality of life. 

In line with Neal et al. [46], this research supports the idea that the tourism industry provides ‘experiences that offer enduring types of satisfaction that positively impact the overall quality of life of those participating in the tourism experience’ (p. 162). These results are also in accordance with Luo et al.’s [18] findings in wellness tourism.

The proposed model also demonstrates the relationship between experience satisfaction and loyalty in accordance with general marketing literature [52] and similar studies [40,41,42]. Following other authors [54,55], this work challenges the traditional conceptualisation of loyalty by considering two objects towards which loyal behaviours can be prompted: the destination and the kind of experience itself. Under this original approach, this research confirms the direct impact that experiential satisfaction exerts on both experiential loyalty (H3+) (β = 0.545 ***) and on destination loyalty (H4+) (β = 0.707 ***). 

This study also finds support for the relationship between quality of life and loyalty, which is in line with the outcomes of other authors [26,54,59], validating the importance of quality of life as an effective predictor of loyalty behaviours. However, some specifications are needed in the context of this research. The direct impact that quality of life has on destination loyalty is low (H6+) (β = 0.135 *) if compared with the one it exerts on experiential loyalty (H5+) (β = 0.346 ***). Then, it can be affirmed that in experiential contexts with healthy water-based tourism, the perceived enhancement of quality of life is a more effective driver of loyal intentions towards the experience rather than for loyalty towards the destination. Therefore, experiences appear to be more valuable tools than destinations in the loyalty formation process, which is possibly even more noticeable in experiences that have the potential of enhancing an individual’s health. 

The positive results obtained by the proposed model support the suitability of considering healthy water-based tourism experiences as a strategy which capitalises water in a sustainable way and fosters economic and social benefits. The former are achieved with the creation of an innovative tourism proposal capable of diversifying the offer of a territory that counts with bodies of water that have marked the landscapes and lifestyles. This diversification of the tourism industry has a positive direct impact on economic revenues and employment. The latter, that is, are social benefits that are referred to two beneficiaries: tourists and residents. Travellers directly benefit from the contact of a new tourism attraction that provides them physical and mental wellbeing and recovery. Indirectly, residents can enjoy the network of infra-structures and services developed for the water-based tourism activity, even more important in rural and depopulated settings. Thus, quality of life is not only promoted for tourists, but also for the residents of the territories in which the tourism activity is developed. 

In addition, it is essential to take into account the development of this type of destination and tourism products from a structured planning point of view. In tourism it is vital to plan bearing in mind the application of sustainable criteria, which is more important in fragile settings such as the ones with water resources. This is one of the most important requirements to implement a successful tourism development strategy in the long term. 

## 6. Conclusions

In this work we explore the impact that tourism water-based activities practiced with a healthy purpose have on significant marketing outcomes such as satisfaction, loyalty and individuals’ quality of life. By applying the 4Es’ model [20], the dimensions of entertainment and esthetics were the most influential in creating experiences in the context considered for this study. As other authors did before in different contexts [27,28,29], this research validated, for the first time, the 4Es’ model in water-and-health tourism.

This work offers an original perspective on how tourism experiences based on water and health can be considered and implemented as a strategy. The main contribution of the study is the confirmation that water-based practices offer experiential value to tourists and exert a positive impact on tourists’ quality of life, satisfaction and loyalty. Those results open a wide ground field of study where water is the central resource supporting new proposals. Water-based experiences can be considered the seed of a new value for water, which in turn can develop and commercialise healthy water-based tourism experiences that can foster economic and social benefits. Given that, the promotion of this new water culture through tourism could enhance the consciousness about the importance of implementing smart and sustainable water management strategies in order to assure the preservation of this essential resource. 

The theoretical contributions of this work are threefold. Firstly, it is confirmed that tourism activities based on water and health are perceived as tourism experiences, according to the 4Es’ model [20], whose scale has been validated in the context of this research. Secondly, the results achieved offer empirical support to the structural model proposed which suggests that the experiences based on water and health have a positive impact on satisfaction, quality of life and loyalty. Finally, this research puts forward a brand-new approach for satisfaction and loyalty. These two variables are studied towards the tourism experience itself, rather than measuring the tourists’ satisfaction with regard to functional elements and loyalty towards the destination. 

The results of this research have useful practical implications for those companies and destinations that have in water and health their main tourism attractions and that wish to turn their offers into more experiential proposals. The study suggests that, in order to foster experientiality in water-based activity, it would be recommendable to put forward tourism products that combine the visits to thermal spas and treatments with other offers, such as nature-based activities, wellness practices (e.g., yoga and mindfulness) and experiences focused on raising a new awareness for the benefits of water on human health.

The study’s findings show how tourism products linking water and health are a suitable response to the current desires and needs of modern tourists, increasingly interested in living authentic, educational and emotional experiences during their holiday time [1,7]. Moreover, this research confirms the important role that water-based experiences have on enhancing the tourists’ perceptions of quality of life, and how this drives tourists’ satisfaction and future loyal behaviours, with a special emphasis on behavioural intentions towards a specific kind of experience (water-based in this context). This suggests that, in the current experiential trend, managers and practitioners need to pursue loyal clients by focusing on the promotion of experiences more than the destination’s attributes in order to better their performances and increase their revenues.

In the context of natural settings, the quest for loyalty from tourists who are interested in healthy water-based tourism experiences has to be interpreted as a sustainable strategy to manage water in the long-term. If healthy water-based tourism experiences were promoted from a natural resources point of view, noticeable benefits for nature, individuals, companies and local residents could be obtained through quality of life and economic and social benefits, which can definitively endorse preserving natural environments and fostering a ‘water culture’. 

Finally, the use of bodies of water for tourism purposes generates a net of interests for the protection of water’s quality not just for its environmental value, but also for being economically worthy as the engine of a new economic and social push. Water-based experiences have the power of revitalising rural economies, generating new employment opportunities, saving decaying societies and, most importantly, encouraging a respectful and long-lasting use of water. 

The limitations and delimitations of this study have to be seen in the use of a non-probability sampling procedure, which could limit the results’ generalisability. The combination of a paper-based and an online technique for data collection may have introduced some bias, even though it did not compromise the validity of the results according to the outputs of a t-test performed. Despite these limitations, this research can possibly contribute to the identification of a new research line linking water and tourism that can add new knowledge to the experience and wellness tourism literature. 

The study was applied in thermal spas and similar establishments. Future research could be focused on nature-based activities related to water, which may offer more consistent results from the application of the 4Es’ model [20]. This may allow a greater generalisation of this study’s results and provide significant contributions to other kinds of destinations and companies that use water as a main tourism attraction.

## Figures and Tables

**Table 1:** Scales used.

Constructs	Authors	Dimensions	Indicators
			This water-based tourism experience…
*Experience*	Song et al. [23] based on Pine and Gilmore [20]	Entertainment	ENT1_…is fun.
			ENT2_…is enjoyable.
			ENT3_…is entertaining.
			ENT4_…is interesting.
		Educational	EDU1_…makes me more knowledgeable.
			EDU2_…is educational.
			EDU3_…allows me to learn more about its benefits for health.
		Escape	ESC1_…allows me to forget my daily routine.
			ESC2_…allows me to have a break from the routine.
			ESC3_…gives me a chance to see myself in a new way.
		Esthetics	EST1_…is attractive.
			EST2_…is pleasant.
			EST3_…is appreciable.
			EST4_…allows me to harmonize myself with the environment.
*Quality of life*	Sirgy et al. [47]	-	QOL1_…has improved my quality of life.
			QOL2_…has made me feel more satisfied with my life.
			QOL3_…makes me feel good about my life, even though I have my ups and downs.
			QOL4_…has enriched my life.
			QOL5_…makes me feel happy.
*Experience satisfaction*	Kim et al. [26]	-	ESA1_My overall evaluation of this water-based tourism experience is positive.
			ESA2_My overall evaluation of this water-based tourism experience is favourable.
			ESA3_I am satisfied with this water-based tourism experience.
			ESA4_I am pleased with this water-based tourism experience.
*Experience loyalty*	Mechinda et al. [62]	-	ELO1_I am loyal to water-based tourism experiences.
			ELO2_My next trip will include some water-based tourism experience.
			ELO3_I would choose a water-based tourism experience again.
			ELO4_I would recommend these water-based tourism experiences to people who seek my advice.
			ELO5_I would tell others positive things about these water-based tourism experiences.
*Destination loyalty*	Kim et al. [26]	-	DLO1_I would recommend to others this destination.
			DLO2_Revisiting this destination would be worthwhile.
			DLO3_I will revisit this destination.
			DLO4_I would like to stay more days in this destination.

**Table 2:** Socio-demographic sample profile (*n* = 184).

Variable	Indicator	Frequency	Percentage
*Gender (n = 174)*	Man	68	37.0
	Woman	106	57.6
*Age (n = 181)*	18–25	4	2.2
	26–35	13	7.1
	36–45	23	12.5
	46–55	31	16.8
	>55	110	59.8
*Place of residence (n = 180)*	Extremadura	74	40.2
	Other region	104	56.5
	Other country	2	1.1
*Level of studies (n = 179)*	Basic	52	28.3
	Secondary	59	32.1
	Higher	67	36.4
	Other	1	0.5
*Frequency of engaging in tourism healthy water-based experiences (n = 154)*	Never	0	0.0
	Hardly ever	24	13.0
	Sometimes	63	34.2
	Frequently	57	31.0
	Always	10	5.4

**Table 3:** Opinion about water-based experiences.

Variable	Indicator	Valid	Mean	Standard Deviation
*Interest in water-based experiences*	Visiting health spas	180	3.87	1.144
	Observing landscapes related to water	167	3.68	1.014
	Visiting fluvial beaches	169	3.41	1.157
	Trekking routes related to water	163	3.35	1.312
	Visiting spas	155	3.09	1.276
	River boat trips	161	2.86	1.284
	Watching aquatic birds	162	2.59	1.278
	Asking for water menus in a restaurant	164	1.87	1.275
*Benefits that water-based tourism experiences have on health*	Benefits that water-based tourism experiences have on health	173	4.24	0.777
	Interest in including water-based experiences in your trip	172	4.01	0.769
	To what extent the possibility of improving your health has motivated your trip	174	4.01	0.968

**Table 4:** Descriptive statistics and measurement model assessment: reflective indicators (I).

Constructs, Dimensions and Indicators	Loadings	*T*-test ^a^	CR ^b^	AVE ^c^
Entertainment_ENT			0.9221	0.7477
ENT1	0.8632 ***	38.6938		
ENT2	0.8471 ***	29.5626		
ENT3	0.9017 ***	53.2662		
ENT4	0.8455 ***	29.2082		
Educational_EDU			0.9049	0.7602
EDU1	0.8757 ***	35.9485		
EDU2	0.8721 ***	30.369		
EDU3	0.8679 ***	35.9734		
Escape_ESC			0.9174	0.7874
ESC1	0.8638 ***	33.494		
ESC2	0.9081 ***	50.922		
ESC3	0.8896 ***	55.7289		
Esthetics_EST			0.9057	0.7073
EST1	0.8911 ***	45.4112		
EST2	0.8781 ***	44.5342		
EST3	0.8606 ***	36.803		
EST4	0.7235 ***	14.1214		
Quality of life_QOL			0.9372	0.7493
QOL1	0.8202 ***	25.7518		
QOL2	0.8949 ***	55.2538		
QOL3	0.8903 ***	49.7992		
QOL4	0.8889 ***	41.3309		
QOL5	0.8306 ***	39.3583		
Experience Satisfaction_ESA			0.9521	0.8324
ESA1	0.9104 ***	51.7311		
ESA2	0.9226 ***	62.5921		
ESA3	0.9202 ***	59.7925		
ESA4	0.8962 ***	50.0434		
Experience loyalty_ELO			0.9132	0.6788
ELO1	0.795 ***	25.0287		
ELO2	0.7272 ***	17.7382		
ELO3	0.8568 ***	34.9623		
ELO4	0.887 ***	45.8576		
ELO5	0.8439 ***	28.8039		
Destination loyalty_DLO			0.9343	0.7815
DLO1	0.873 ***	31.6865		
DLO2	0.9356 ***	73.7108		
DLO3	0.9385 ***	88.5338		
DLO4	0.7796 ***	23.7838		

Note: ^a^ Critical *t*-values: * *p* < 0.05; ** *p* < 0.01; *** *p* < 0.001; ns not significant (based on *t*(4999), one-tailed test); *t*(0.05; 4999) = 1.645; *t*(0.01; 4999) = 2.327; *t*(0.001; 4999) = 3.092. ^b^ Composite reliability. ^c^ Average variance extracted.

**Table 5:** Discriminant validity assessment (I).

	QOL ^a^	EDU ^b^	ENT ^c^	EST ^d^	ESC ^e^	DLO ^f^	ELO ^g^	ESA ^h^
**QOL**	**0.8656**							
**EDU**	0.6249	**0.8719**						
**ENT**	0.6695	0.5789	**0.8647**					
**EST**	0.7201	0.5713	0.7306	**0.8410**				
**ESC**	0.7015	0.4681	0.6451	0.7592	**0.8874**			
**DLO**	0.6141	0.4417	0.5531	0.6178	0.5842	**0.8840**		
**ELO**	0.715	0.5435	0.6039	0.6674	0.5689	0.7397	**0.8239**	
**ESA**	0.6783	0.4157	0.6493	0.6772	0.6002	0.7983	0.779	**0.9124**

^a^ Quality of life. ^b^ Educational. ^c^ Entertainment. ^d^ Esthetics. ^e^ Escape. ^f^ Destination loyalty. ^g^ Experience loyalty. ^h^ Experience Satisfaction.

**Table 6:** Measurement model assessment: reflective indicators (II).

Constructs and Indicators	Loadings	*T*-test^a^	CR ^b^	AVE ^c^
Quality of life_QOL			0.9372	0.7493
QOL1	0.8202***	25.3672		
QOL2	0.8949***	55.3579		
QOL3	0.8903***	49.699		
QOL4	0.8889***	40.5309		
QOL5	0.8306***	39.3498		
Experience Satisfaction_ESA			0.9521	0.8324
ESA1	0.9104***	52.6669		
ESA2	0.9226***	64.0527		
ESA3	0.9202***	59.8218		
ESA4	0.8962***	50.4753		
Experience loyalty_ELO			0.9132	0.6788
ELO1	0.795***	25.3992		
ELO2	0.7272***	17.9474		
ELO3	0.8568***	35.195		
ELO4	0.887***	45.2408		
ELO5	0.8439***	28.4732		
Destination loyalty_DLO			0.9343	0.7815
DLO1	0.873***	31.3706		
DLO2	0.9356***	74.7519		
DLO3	0.9385***	87.8543		
DLO4	0.7796***	22.701		

Note: ^a^ Critical *t*-values: * *p* < 0.05; ** *p* < 0.01; *** *p* < 0.001; ns not significant (based on *t*(4999), one-tailed test); *t*(0.05; 4999) = 1.645; *t*(0.01; 4999) = 2.327; *t*(0.001; 4999) = 3.092. ^b^ Composite reliability. ^c^ Average variance extracted.

**Table 7:** Discriminant validity assessment (II).

	QOL ^a^	DLO ^b^	ELO ^c^	ESA ^d^
**QOL**	**0.8656**			
**DLO**	0.6141	**0.8840**		
**ELO**	0.715	0.7397	**0.8239**	
**ESA**	0.6783	0.7983	0.779	**0.9124**

^a^ Quality of life. ^b^ Destination loyalty. ^c^ Experience loyalty. ^d^ Experience Satisfaction.

**Table 8:** Collinearity statistics and analysis of formative indicators.

Construct	Indicators (scores)	VIF^a^	Weight	*T*-test ^b^ (weight)	Loading	*T*-test^b^ (loading)
**Experience**	EDU ^c^	1.632	−0.0566 ^ns^	0.5567	0.5772 ***	7.133
	ENT ^d^	2.441	0.4425 ***	3.4934	0.9016 ***	22.3293
	EST ^e^	3.313	0.5034 ***	3.1069	0.9404 ***	28.3335
	ESC ^f^	2.581	0.1923 ^ns^	1.4636	0.8335 ***	15.9033

^a^ Variance inflation factor. ^b^ Critical *t*-values: * *p* < 0.05; ** *p* < 0.01; *** *p* < 0.001; ns not significant (based on *t*(4999), two-tailed test); *t*(0.05; 4999) = 1.65; *t*(0.01; 4999) = 1.96; *t*(0.001; 4999) = 2.58. ^c^ Educational. ^d^ Entertainment. ^e^ Esthetics. ^f^ Escape.

**Table 9:** Effects on endogenous variables and structural model results.

Hypotheses	R^2a^	Direct Effect (β)	Correlation	Explained Variance	*T*-test (bootstrap)^b^	Supported
Experience satisfaction	0.5186			51.9%		Yes
H1+: Experience→ Experience satisfaction		0.7201 ***	0.7201	51.9%	16.5828	Yes
Quality of life	0.4601			46.0%		Yes
H2+: Experience satisfaction→ Quality of life		0.6783 ***	0.6783	46.0%	14.49	Yes
Experience loyalty	0.6713			67.1%		Yes
H3+: Experience satisfaction→ Experience loyalty		0.5445 ***	0.779	42.4%	9.2554	Yes
H5+: Quality of life→ Experience loyalty		0.3457 ***	0.715	24.7%	5.541	Yes
Destination loyalty	0.6471			64.7%		Yes
H4+: Experience satisfaction→ Destination loyalty		0.7071 ***	0.7983	56.4%	11.8028	Yes
H6+: Quality of life→ Destination loyalty		0.1346 *	0.6141	8.3%	2.1557	Yes

Notes: ^a^ R^2^ value of 0.75, 0.5 or 0.25 for the latent endogenous variables in structural models can be considered substantial, moderate or weak, respectively [61]. ^b^ Critical *t*-values: * *p* < 0.05; ** *p* < 0.01; *** *p* < 0.001; ns not significant (based on *t*(4999), one-tailed test); *t*(0.05; 4999) = 1.645; *t*(0.01; 4999) = 2.327; *t*(0.001; 4999) = 3.092.
